# Global analyses of *Ceratocystis cacaofunesta* mitochondria: from genome to proteome

**DOI:** 10.1186/1471-2164-14-91

**Published:** 2013-02-11

**Authors:** Alinne Batista Ambrosio, Leandro Costa do Nascimento, Bruno V Oliveira, Paulo José P L Teixeira, Ricardo A Tiburcio, Daniela P Toledo Thomazella, Adriana F P Leme, Marcelo F Carazzolle, Ramon O Vidal, Piotr Mieczkowski, Lyndel W Meinhardt, Gonçalo A G Pereira, Odalys G Cabrera

**Affiliations:** 1 Laboratório de Genômica e Expressão, Departamento de Genética Evolução e Bioagentes, Instituto de Biologia, Universidade Estadual de Campinas, CEP: 13083-970, Campinas, São Paulo, Brasil; 2 Laboratório Nacional de Biociências-LNBio, Centro Nacional de Pesquisa em Energia e Materiais, CEP: 13083-970, Campinas, São Paulo, Brasil; 3 Centro Nacional de Processamento de Alto Desempenho, Universidade Estadual de Campinas, CEP: 13083-970, Campinas, São Paulo, Brasil; 4 High-Throughput Sequencing Facility, University of North Carolina, 2234 Nelson Highway, Chapel Hill, NC, NC 27516, USA; 5 Sustainable Perennial Crops Laboratory, USDA/ARS, 10300 Baltimore Ave, Beltsville, MD, 20705, USA

**Keywords:** Cacao wilt disease, *Ceratocystis cacaofunesta*, Mitogenomics, Mitochondrial proteome, Fungal virulence

## Abstract

**Background:**

The ascomycete fungus *Ceratocystis cacaofunesta* is the causal agent of wilt disease in cacao, which results in significant economic losses in the affected producing areas. Despite the economic importance of the *Ceratocystis* complex of species, no genomic data are available for any of its members. Given that mitochondria play important roles in fungal virulence and the susceptibility/resistance of fungi to fungicides, we performed the first functional analysis of this organelle in *Ceratocystis* using integrated “omics” approaches.

**Results:**

The *C. cacaofunesta* mitochondrial genome (mtDNA) consists of a single, 103,147-bp circular molecule, making this the second largest mtDNA among the Sordariomycetes. Bioinformatics analysis revealed the presence of 15 conserved genes and 37 intronic open reading frames in *C. cacaofunesta* mtDNA. Here, we predicted the mitochondrial proteome (mtProt) of *C. cacaofunesta*, which is comprised of 1,124 polypeptides - 52 proteins that are mitochondrially encoded and 1,072 that are nuclearly encoded. Transcriptome analysis revealed 33 probable novel genes. Comparisons among the Gene Ontology results of the predicted mtProt of *C. cacaofunesta*, *Neurospora crassa* and *Saccharomyces cerevisiae* revealed no significant differences. Moreover, *C. cacaofunesta* mitochondria were isolated, and the mtProt was subjected to mass spectrometric analysis. The experimental proteome validated 27% of the predicted mtProt. Our results confirmed the existence of 110 hypothetical proteins and 7 novel proteins of which 83 and 1, respectively, had putative mitochondrial localization.

**Conclusions:**

The present study provides the first partial genomic analysis of a species of the *Ceratocystis* genus and the first predicted mitochondrial protein inventory of a phytopathogenic fungus. In addition to the known mitochondrial role in pathogenicity, our results demonstrated that the global function analysis of this organelle is similar in pathogenic and non-pathogenic fungi, suggesting that its relevance in the lifestyle of these organisms should be based on a small number of specific proteins and/or with respect to differential gene regulation. In this regard, particular interest should be directed towards mitochondrial proteins with unknown function and the novel protein that might be specific to this species. Further functional characterization of these proteins could enhance our understanding of the role of mitochondria in phytopathogenicity.

## Background

*Ceratocystis cacaofunesta* is an ascomycete fungus (Class Sordariomycetes, Order Microascales) that causes wilt disease in cacao (*Theobroma cacao*). This fungus is a member of the Latin American clade of the *Ceratocystis fimbriata* complex [1,2], a taxonomic group that includes species with high genetic variability and wide host ranges [3,4]. These species cause canker and wilt diseases in many economically important crops, such as *Coffea arabica* and *Eucalyptus spp.*[5]. Engelbrecht and Harrington [6] proposed that host specialization may influence speciation in this group.

*C. cacaofunesta* is indigenous to Central and South America [1,2]. In Brazil, this pathogen was first reported in the Amazon region [7]. In the 1990s, *C. cacaofunesta* was introduced to the southern region of Bahia, which is the largest Brazilian cacao-producing state [8]. This fungus is able to penetrate cacao trees through stem wounds that are caused either by insects (natural vectors) or through infected cutting tools [9]. Unlike other cacao diseases that primarily affect branches and fruits, such as Witches’ broom disease and Frosty pod, wilt disease is a systemic infection that damages the entire plant. The fungus enters its host through the secondary xylem, resulting in the formation of deep spots and leading to the obstruction of water and nutrient transport [10]. Consequently, the plant turns yellow and then brown; the infection culminates in the wilting and sudden death of the tree. This disease is responsible for reductions in the cacao population in plantation areas, which has resulted in great economic losses in the affected regions. Extensive damages have also been reported in Trinidad [11], Venezuela and Colombia [12].

Several studies have attempted to characterize the genetic variation, aggressiveness and host specialization of different populations of *C. fimbriata complex* including *C cacaofunesta*[6,13-15]. However, little is known at the molecular level regarding this fungus and its interaction with cacao. Notably, no genomic data are available for any members of the *Ceratocystis* genus. In 2011, our group initiated the *C. cacaofunesta* Genome Project (http://www.lge.ibi.unicamp.br/ceratocystis), with the goal of understanding the mechanisms that underlie the interactions between *C. cacaofunesta* and *Theobroma cacao* in the development of wilt disease. We initially focused on the study of *C. cacaofunesta* mitochondria for two reasons: (i) their potential role in fungal pathogenesis and (ii) the relevance of this organelle as a target for fungicides [16].

In addition to their canonical function as an energy-producing compartment, mitochondria are involved in multiple cellular processes [17]. These organelles play important roles in calcium homeostasis [18], the biosynthesis of iron-sulfur clusters [19], lipid and amino acid metabolism, aging and the signaling of programmed cell death [20]. Due to the importance of these processes, mitochondrial dysfunctions cause serious consequences for the cell and, ultimately, the entire organism. Functionally compromised mitochondria are associated with senescence in non-pathogenic fungi, such as *Podospora anserina*[21] and *N. crassa*[22].

Mitogenomics has become a useful tool for evolutionary studies [23], and the continuous advances in this field have contributed to the understanding of the diverse topology, organization and structure of mtDNA in fungi [24]. However, as mtDNA encodes approximately 1% of the mitochondrial proteome, scant information concerning the roles of mitochondria in the metabolism, development and lifestyle of organisms has been gleaned from exclusive analyses of the mitochondrial genome. Fungal mtDNA generally contains 14 genes that encode hydrophobic subunits of the respiratory chain complexes, two genes that encode ribosomal RNAs (small and large subunits) and genes that encode a full set of tRNAs [25]. The other 99% of the mitochondrial proteome is encoded by nuclear DNA and imported into the mitochondria [26]. Nuclear-encoded mitochondrial proteins (NMP) are produced by cytosolic ribosomes and are targeted to the proper mitochondrial subcompartment [27-29].

To understand mitochondrial function, studies using genomic, transcriptomic and proteomic approaches have been performed in several types of organisms, such as fungi [30], plants [31] and humans [32]. The first mitochondrial analysis using integrative proteomic and genomic approaches in fungi was conducted in *S. cerevisiae*[33]. By overcoming the individual limitations of each technique, this integrative study produced a powerful tool for the prediction of mitochondrial processes in yeast. The *S. cerevisiae* mitochondrial proteome contains an estimated 1,000 proteins, of which 851 were identified using proteomic assays [34]. In *N. crassa*, proteomic studies have led to the identification of 438 mitochondrial proteins [35]. Proteomics has previously been used to illuminate central processes in phytopathogenic fungi [36,37]. Certain more recent studies have analyzed of mycelial, conidiospores and haustoria proteomes [37-41], fungal secretomes and proteomes that are associated with fungal virulence [42]. However, the mitochondrial proteomes of phytopathogenic fungi have not been explored.

In the present study, we performed a global analysis of *C. cacaofunesta* mitochondria using an integrative approach (mitogenomics, transcriptomics and proteomics). We predicted the *C. cacaofunesta* mitochondrial proteome (mtProt), including mitochondrial-encoded proteins and mitochondrial proteins encoded in the nuclear genome (NMP). Moreover, 27% of predicted mtProt was validated by experimental proteome analysis (LC-MS/MS). We focused on comparisons with available mitochondrial proteomes from non-pathogenic model fungi to increase our knowledge on the role of mitochondria in pathogenicity. The present study is the first partial genomic analysis of a *Ceratocystis* species to be published. Additionally, to the best of our knowledge, this study is pioneering in that it presents a global analysis of the mitochondrial proteome of a phytopathogenic fungus using this integrative approach.

## Results and discussion

### *C. cacaofunesta* mitochondrial genome structure

**Figure 1:**
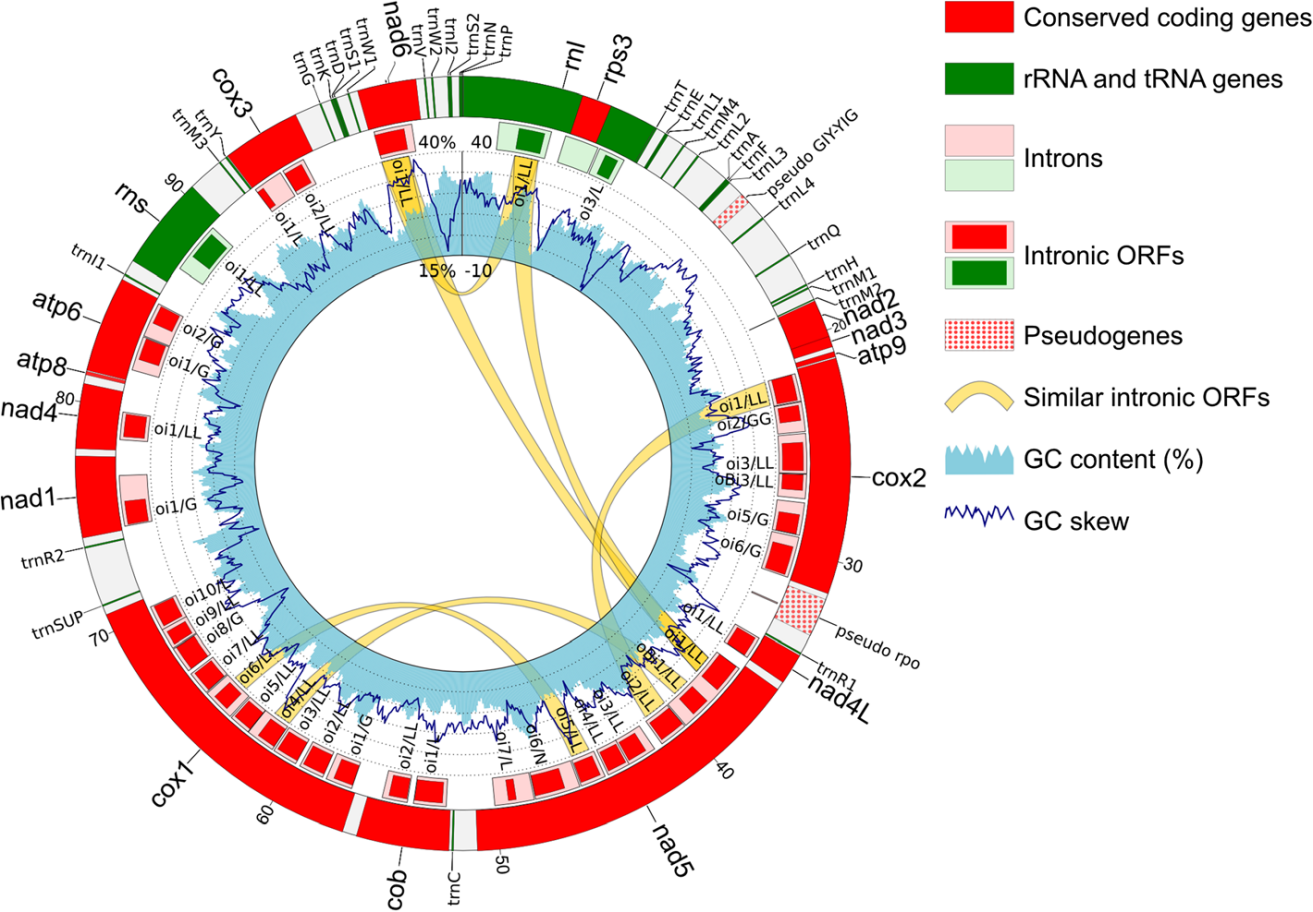
**Map of *C*. *cacaofunesta *mitochondrial DNA.** The numbers along the outermost circle are the DNA coordinates in kb, beginning at 12 o’clock and continuing clockwise. The scales for the GC content (in %, light blue histogram) and the GC skew (dimensionless, black curve) are at 12 o’clock in the innermost circle. Conserved coding genes (reds), pseudogenes (red dots), tRNAs and rRNAs (green) are shown in the outermost circle. Introns (light red) and intronic ORFs (red) are shown in the middle circle. The intronic ORFs of each gene are numbered as *oi* (intron number). The domains of each intronic ORF are indicated as LL (for LAGLIDADG) or G (for GIY-YIG). Similar intronic ORFs (with more than 50% coverage and identity) are linked by yellow ribbons.

The complete mitochondrial genome of *Ceratocystis cacaofunesta* was assembled with 2,153-fold coverage as a single circular molecule comprising 103,147 bp (Figure 1). It is the fourth largest published fungal mitochondrial genome, and the second largest among the Sordariomycetes, after that of *Chaetomium thermophylum* (127 kb) [43]. However, the differences in the sizes of fungal mitochondrial genomes are not correlated with differences in their number of conserved genes [44,45]. Therefore, *C. cacaofunesta* mtDNA contains 15 genes that encode the conserved proteins NADH dehydrogenase subunits 1 to 6 and 4 L (*nad*1 to nad6 and nad4 L); cytochrome c oxidase subunits I, II and III (*cox*1, *cox*2 and *cox*3); ATP synthase subunits 6, 8 and 9 (*atp6, atp8* and *atp9*); apocytochrome b (*cob*); and the ribosomal protein S3 (*rps3*), (Figure 1). With the exception of *rps3*, all of these conserved genes are involved in oxidative phosphorylation and ATP synthesis. Genes that encode the small and large rRNA subunits (*rns* and *rnl*, respectively) were also identified. The *rps3* gene is located within a group I intron (small RNAs that have ability to self-splice from RNA transcripts) of the *rnl* gene, as has been described for many other Sordariomycetes [46,47]. It has been proposed that the structure of one gene (e.g., *rps3*) within another (e.g., *rnl*) guarantees that they are co-transcribed and that the stoichiometry of the two components is adequate for ribosome biogenesis [46]. In addition to rRNA and conserved coding genes, the tRNAscan-SE program [48] identified 31 putative tRNAs for the 20 standard amino acids and a possible suppressor tRNA (amber tRNA). All of the tRNAs, rRNAs and protein-coding genes are oriented in the same direction (clockwise in Figure 1). Also, the conserved genes are organized in the same four synthetics units described for *Sordariomycetes*[49], except by minor changes in *trn* genes distribution. The gene pair *nad2*-*nad3* is partially overlapping as the *nad2* ORF extends 49 nucleotides into the *nad3* reading frame (Figure 1). It has been proposed that the preservation of syntenic units could play a functional role, enabling the polycistronic transcription of these genes [49].

The annotation of the *C. cacaofunesta* mt genome revealed that introns form 48.7% of this DNA molecule, explaining its large size. The invasion of mitochondrial genes by group I introns is a primary reason for the wide variation in fungal mitochondrial genome sizes (Additional file 1) [50]. A total of 37 mitochondrial group I introns, with an average size of 1,535 bp, were identified in the conserved coding and rRNA genes. These introns are distributed within conserved genes throughout the *C. cacaofunesta* mitochondrial genome, with *cox1* harboring the largest number of these elements (10 introns) (Figure 1, Additional file 1). *Cox*1 has been described as a reservoir for mitochondrial group I introns in fungi, harboring as many as 18 of these elements in *Agaricus bisporus*[51]. Of the total of mitochondrial group I interns, 36 encoded homing endonuclease genes (HEG) (Figure 1, Additional file 1). The dynamic properties of these introns suggest that they can be moved into other regions of the genome and between the genomes of phylogenetically distant species [52].

Although the presence of mitochondrial plasmids is a common feature of Sordariomycetes, such as *G. zeae*[53], *N. crassa*[54]*and P. anserine*[55], no integrated or free linear mitochondrial plasmids were identified in the *C. cacaofunesta* mtDNA. However, a pseudogene with similarity to the RNA polymerase (*rpo*) of the *P. anserina* mitochondrial plasmid pAL2-1(e-value: 4e^-92^, 100% of query coverage and 57% of positives matches) was identified downstream of the *cox1* gene (Figure 1, labeled, “pseudo *rpo*”). The presence of this pseudogene with plasmidial origin suggests an ancestral plasmid insertion in this region. We found no evidence of inverted repeats flanking this pseudogene.

The analysis of the *C. cacaofunesta* mtDNA also revealed an average of 26.8% G+C residues, which were uniformly distributed throughout the sequence (Figure 1, blue histogram). The intronic ORFs exhibited a slightly lower GC content (25.2%) than did the conserved genes (26.5%). Moreover, a GC skew analysis was performed, and the shift points of the GC skew graphs were consistent with the loci of the origin (*org*) and termination *(ter)* of replication in bacteria [56] and certain fungi [57].

### Predicted mitochondrial proteome and annotation

Considering that approximately 99% of the mitochondrial proteins are encoded in the nucleus [25] and that we have the complete genome sequences of *C. cacaofunesta*, we estimated the total mitochondrial proteome of *C. cacaofunesta* based on the numbers of mitochondrial- and nuclear-encoded proteins.

**Figure 2:**
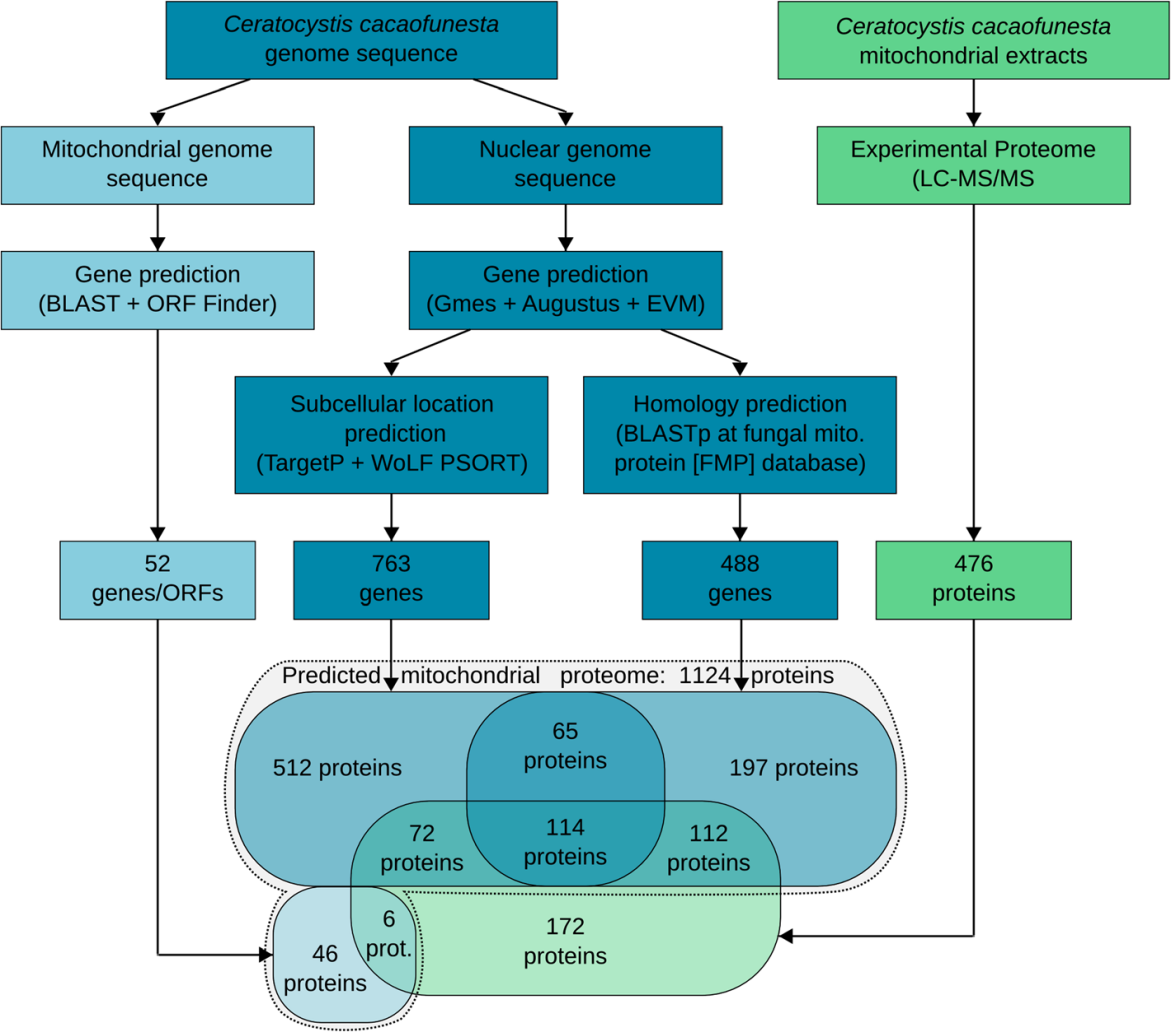
**A Flowchart of the mitochondrial proteome prediction (blue) and experimental proteome (green).** The complete set of predicted mitochondrial proteins is surrounded by a black dotted line.

The use of these combined approaches generated a predicted mitochondrial proteome for *C. cacaofunest*a that consisted of 1,124 polypeptides, with 1,072 NMP and 52 mitochondrial-encoded proteins. Of the total proteins, 584 were identified using the predictor softwares (TargetP and WoLF PSORT), 309 were identified only by homology analysis and 179 were identified using both methods (Figure 2, Venn diagram). The predicted *C. cacaofunesta* mtProt has a similar size to the mtProt that was estimated for *S. cerevisiae* (approximately 1,000 polypeptides) [58]; whereas the *A. thaliana* mtProt is estimated to contain approximately 850 polypeptides [31], and the mouse mtProt contains about 1,500 polypeptides [59].

The predicted mtProt of *C. cacaofunesta* was annotated using AutoFACT [60] to summarize the results of the BLAST [61] searches of the NR/NCBI, KEGG [62] and UniRef90 databases [63]. An independent search of the CDD (conserved domain) database [63] was also performed. The Blast2GO [64] program was used to produce Gene Ontology (GO) classification (see Material and Methods). Table 1 summarizes the main results of annotation. From the predicted MtProt, 38 codifying genes result in no hits in AutoFACT. These genes were collectively annotated as hypothetical unconserved genes. Three hundred forty-nine other genes were similar only with genomic sequences and/or predict hypothetical proteins; these genes were therefore annotated as hypothetical conserved genes. Lastly, the remaining 737 genes are similar to at least one other known (described) gene in the databases that were used for AutoFACT annotation and were therefore considered to be known conserved genes. The GO annotation assigned 724 proteins to at least one level of ontology (411 proteins were assigned to cellular component, 619 to molecular function and 599 to biological process). A total of 190 enzymes were identified by Blast2GO, from which, 138 were distributed in 78 metabolic pathways. The most represented metabolic pathway were nitrogen metabolism (41 enzymes) and oxidative phosphorylation (40 enzymes). Additional file 2 shows the entire catalogue of predicted *C. cacaofunesta* mitochondrial proteins and their respective annotation.

**Table 1:** Summary of MtProt annotation

**Gene category**	**Predicted MtProt**	**Experimental MtProt**
Hypothetical unconserved	38	1
Hypothetical conserved	349	83
Known conserved	737	220
Total	1,124	304

### Transcriptomic analysis

A global analysis of the *C. cacaofunesta* transcriptome was performed using large-scale mRNA sequencing (RNA-Seq), as described in the corresponding section of Methods. This technique is widely used to analyze gene expression and to validate gene predictions [65-68]. This methodology was recently used to analyze mitochondrial gene expression [69,70]. Approximately 55 million of reads were generated in two biological replicates (CER1 and CER2). As expected, only a very small number of reads mapped to mitochondrial genes (400 from CER1 and 600 from CER2) (Additional file 2A). The RNAseq methodology used here requires a mRNA polyA tail, but RNAs that are transcribed from fungal mtDNAs generally lack this feature. Polyadenylation of mitochondrial mRNA appears to be restricted to higher eukaryotes [71]. Therefore, the 1,000 RNAseq reads aligned with *C. cacaofunesta* mtDNA most likely represents artifacts.

**Figure 3:**
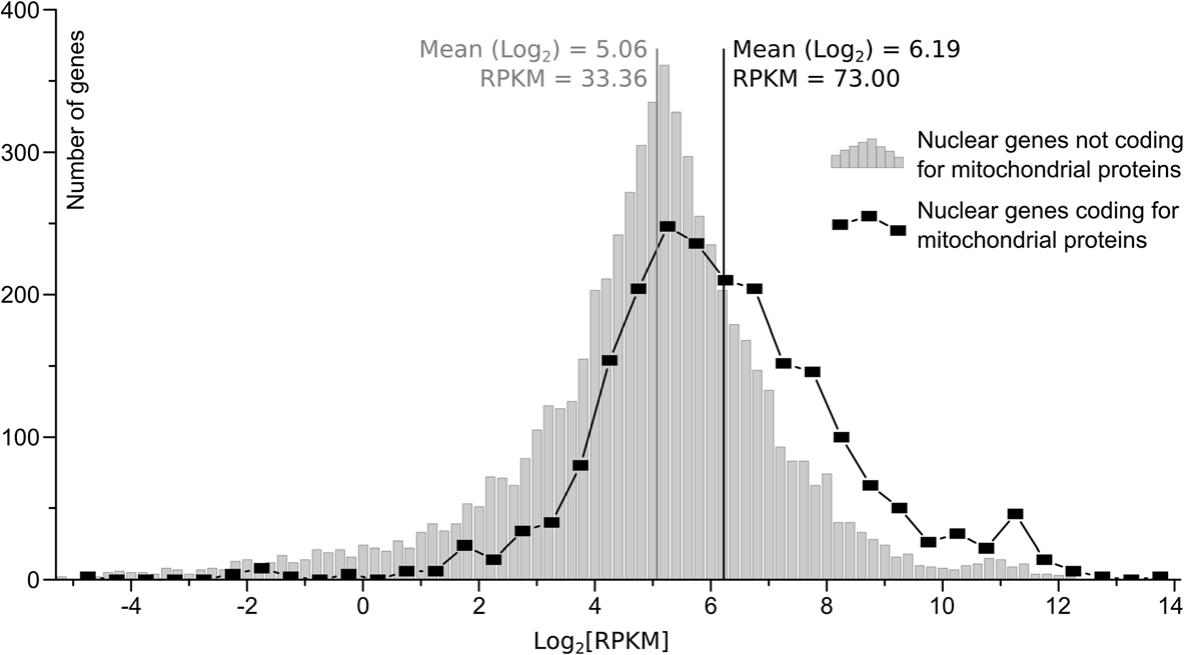
**The distribution of RPKM values (in log**_**2 **_**scale) in mitochondrial (black line) and non-mitochondrial (gray histogram) genes.** The transcription of the mitochondrial genes was 2.18 x higher (on average) than that of the non-mitochondrial genes.

Genes with RPKM < 1 were considered to not be expressed. Among the total reads that mapped to nuclear genes 21.6% (approximately 12 million) align with genes that encode mitochondrial proteins (5.6 and 6.2 million from CER1 and CER2, respectively). This result indicates that the average expression of genes that encode NMPs is approximately 2.18 times greater than the expression of the other nuclear genes (mean RPKMs of 73.00 and 33.36, respectively) (Figure 3). Of the 1,072 NMP genes, only 10 presented RPKM values of less than 1, and 398 had RPKM values greater than 100 (Additional file 2B). More highly expressed genes are involved in protein synthesis and oxidative phosphorylation, which is consistent with the high rates of fungal growth in the culture conditions. Approximately 99% of the NMP codifying genes are expressed. RNAseq also aids in the identification of new genes. Based on our prediction, a total of 38 novel putative genes encode mitochondrial proteins. However, RNAseq analysis indicated that only 33 of these are expressed (RPKM > 1) (Additional file 2B).Transcriptomic information consists of the complete set of transcripts and their abundance in the cell in specific physiological conditions [36]. Therefore, the transcriptome of fungi *in vitro* may be quite different when compared with the transcriptome of fungi during its interaction with its host. Although we are unable to make conclusions regarding expression profile of *C. cacaofunesta* genes during pathogenesis based on these data, the transcriptome analysis of *C. cacaofunesta* mitochondrial genes *in vitro* provides valuable information with respect to validating gene prediction, including the discovery of 33 novel putative genes.

### Experimental mitochondrial proteome

The isolated mitochondrial proteome was electrophoretically separated on a 12.5% SDS-PAGE gel, and 19 bands were excised from the gel and subjected to LC-MS/MS analysis in the corresponding section (Additional file 3). A total of 12,181 MS/MS spectra were generated and confidently assigned to 476 unique proteins with statistically significant (p < 0.05) Mascot-based scores and a 9.1% FDR (Additional file 4, Sheet 4A). Among the 476 proteins that were identified using LC-MS/MS, the 5 most abundant proteins presented more than 140 spectral counts in both samples (Additional file 4, Sheet 4A). The size of these proteins ranged from 34 to 138 kDa, and there was not a molecular weight bias in their abundance.

Among the total proteins that were identified using LC-MS/MS, 64% (304) were contained within the predicted mtProt (Figure 2). This result indicates that approximately 27% of the predicted mtProt was experimentally validated. Accordingly, the mitochondrial proteome analysis by LC-MS/MS is capable of identifying 23%-40% of the known mitochondrial proteins but does not capture low-abundance proteins [59], except for proteins that are produced under specific circumstances [72]. Experimental mitochondrial proteome analysis of *N. crassa* has identified 473 proteins that correspond to 169 unique genes; these represent 20% of the total mtProt that has been described for this species [73].

Of the 304 predicted mitochondrial proteins that were validated by LC-MS/MS, we identified 5 mitochondrial-encoded conserved proteins (ATP6, COB, COX1, COX2 and NAD2) and an intronic ORF (oi5cox2). Table 2 lists a subset of the experimentally identified NMP proteome that was associated with important metabolic process. According with transcriptome analysis, the set of experimentally identified proteins reflects the metabolic status of the fungal cell in rich growth conditions.

**Table 2:** **Proteins identified by LC**-**MS**/**MS involved in the respiration metabolic process**

**Methabolic Process**	**Description**	**Number**
Pyruvate dehydrogenase complex	Pyruvate Dehydrogenase (lipoamide) alpha	1
	Pyruvate Dehydrogenase (lipoamide) beta	1
	Pyruvate Dehydrogenase Kinase	1
	Dihydrolipoamide S-acetyltransferase	1
	Total	4
Tricarboxylic acid cycle	2-methylcitrate dehydratase	2
	Citrate Synthase	2
	Isocitrate Dehydrogenase1	1
	Isocitrate Dehydrogenase 2	1
	Isocitrate dehydrogenase	1
	Homoisocitrate dehydrogenase	1
	Isocitrate lyase	1
	Total	9
Oxidative phosphorylation	Complex I	13
	Complex II	3
	Complex III	3
	Complex IV	2
	Complex V	5
	Total	25

Remarkably, we identified 83 conserved hypothetical proteins and 1 novel protein in the experimental proteome that exhibited putative mitochondrial localization (Additional file 4, Sheet 4B). We believe that these results are an important contribution because they give the first experimental evidence of the existence of these putative mitochondrial proteins. The functional characterization of these proteins and their association with particular mitochondrial pathways is a great challenge but could certainly improve our understanding of this organelle.

We also identified 172 proteins that were not predicted to be mitochondrial based on our analysis (Figure 2, Additional file 4, Sheet 4C). Manual annotation was used to elucidate the composition of this group of proteins. Total number of mitochondrial proteins identified using LC-MS/MS could reach 388 (304 from prediction, and 84 from manual annotation), representing 81% of the total experimental proteome. This result indicates that the enriched-mitochondrial preparation of *C. cacaofunesta* was performed with high efficiency. However, the standard methodology chosen here was automatic annotation due to the high stringency of the parameters; hence, the 84 proteins that were classified as putative mitochondrial based on manual annotation were not included in the final catalogue of mitochondrial proteins that are proposed here. The experimental proteome was used to validate a fraction of predicted proteome, focusing on the identification of hypothetical and novel proteins.

One of the main contributions of this study is the experimental identification of two groups of predicted mitochondrial proteins with no assigned function: (i) proteins for which there was no experimental evidence (hypothetical proteins) and (ii) novel proteins. Table 1 shows the total of proteins of groups (i) and (ii) based both on the predicted and the experimental proteomes. Of the 349 predicted hypothetical proteins, 83 were identified by mass spectrometry, as mentioned above. With respect to group (ii), the predicted mtProt identified 38 novel putative proteins. However, RNAseq confirmed the expression of 33 of them, and one protein was identified experimentally. The 84 hypothetical proteins that were identified by mass spectrometry were reclassified as conserved unknown function (Additional file 4, Sheet 4B). The *S. cerevisiae* mitochondrion is the best-understood and characterized molecularly [24,29,33,72]. However, approximately 19% of the identified proteins remain have no known function [29]. The predicted *C. cacaofunesta* mtProt contains approximately 34% of proteins with unknown function. These data suggest that we are far from fully understanding the function of this organelle, a fact that reflects its functional plasticity. Further functional characterization of these proteins may enable a better understanding of mitochondrial function. It is important to highlight that there are more than 65 known Sordariomycetes genomes published, and we suggest that the 38 predicted novel genes (33 expressed) may be specific to this species. Moreover, it is very likely that these proteins are shared with other species from the *Ceratocystis fimbriata* complex, a hypothesis that will investigated with the upcoming availability of other *Ceratocystis* genomes. Our results provide a framework for examining the involvement of novel proteins in mitochondrial pathways.

### The functional annotation of the *C. cacaofunesta* mitochondrial proteome and a comparison with those of *S. cerevisiae* and *N. crassa*

**Figure 4:**
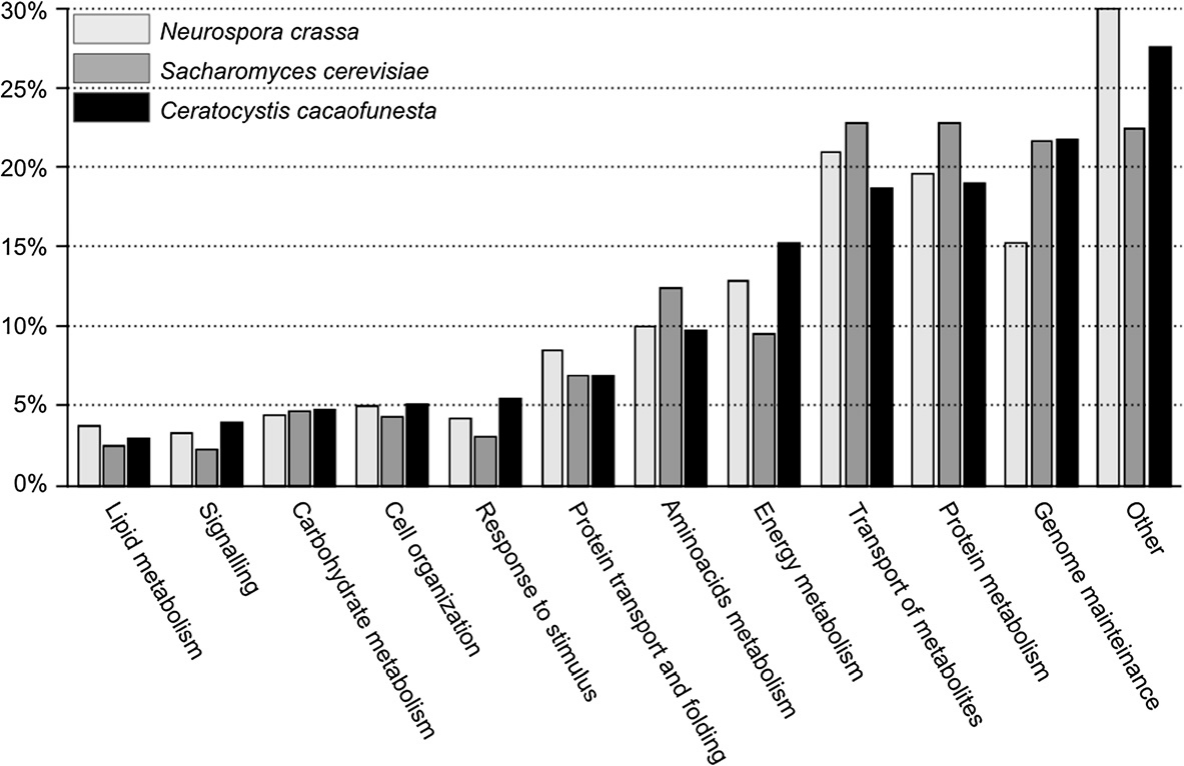
**Distribution of Gene Ontology (GO) categories for the predicted mitochondrial genes of *N*. *crassa *(light gray), *S*. *cerevisiae *(dark gray) and *C*. *cacaofunesta *(black)**.

We focused on the GO annotation to perform a global function analysis of the predicted *C. cacaofunesta* proteome. Putative functions were assigned to the predicted mtProt using GO and were manually grouped into 12 categories according to biological process. Only the amino acid, carbohydrate and lipid metabolism categories were queried using specific GO IDs: GO:0006519, GO:0005975 and GO:0006629, respectively. The remaining 9 categories included several related GO IDs (Additional file 5). The GO distribution is shown in Figure 4, together with the GO distributions of *N. crassa* and *S cerevisiae* mtProt. The largest GO category is “other” (27.6%), which was expected given that it contained proteins from 26 small categories (Additional file 6, Sheet 6A). The second-most represented category is “genome maintenance and transcription”, which includes 21.7% of all of the predicted mitochondrial proteins. Based on sequence, approximately 19% of the mtProt are related to protein metabolism, and 18.7% of them are assigned to “transport of metabolites”. This latter category includes ATP-binding cassette (ABC) transporters, ion transport families, protein transporters and other metabolite transporters. More than 15.2% of the proteins were directly involved in energy metabolism, including parts of the oxidative phosphorylation machinery (OXPHOS), members of the tricarboxylic acid cycle (TCA), and the pyruvate dehydrogenase complex (PDH). Among underrepresented categories were the following: (i) response to stimulus and (ii) signaling, which included 5.5% and 4.0% of all of the predicted mitochondrial proteins, respectively (Figure 4). At 3.0%, lipid metabolism was the smallest category represented in the *C. cacaofunesta* mtProt.

We used the same GO categorization criteria as above to annotate the *S. cerevisiae* and *N. crassa* mitochondrial proteomes [72,73] to compare their global functional profiles to that from *C. cacaofunesta* (Figure 4 and Additional file 6, Sheet 6B and 6C). Due to the redundancy of GO categories associated with a same protein, it is difficult to perform a statistical analysis of these data. However, Figure 4 clearly shows that there is a similar pattern to the distribution of mitochondrial proteins in different GO functional categories among the three species. Considering the important role of mitochondrial function to basic cell metabolism, the conservation of core mitochondrial functions is expected [74]. However, it is known that the expansion or reduction in the size of individual protein families, and hence individual functions, plays a specific role in lifecycle of the organism. In phytopathogenic fungi, differences in protein families have been associated with the importance of the related functions in plant-pathogen interactions [5]. Considering that *C. cacaofunesta* is a phytopathogenic fungus and that *S. cerevisiae* and *N. crassa* are non-pathogenic, it would be plausible to expect more differences between these species regarding the distribution of proteins in functional categories that are related to transport of metabolites, signaling, and defense [75]. However, no such major differences were identified. According the most recent publication on the subject, certain categories that were represented in this analysis, such as signaling, energy metabolism, lipid metabolism, protein transport and protein folding, have similar protein percentages as those that have been described for the *S. cerevisiae* mtProt [29]. These results suggest that the involvement of the mitochondria in virulence and pathogenicity may rely on specific proteins and/or in the convergence in time and space of different components.

**Figure 5:**
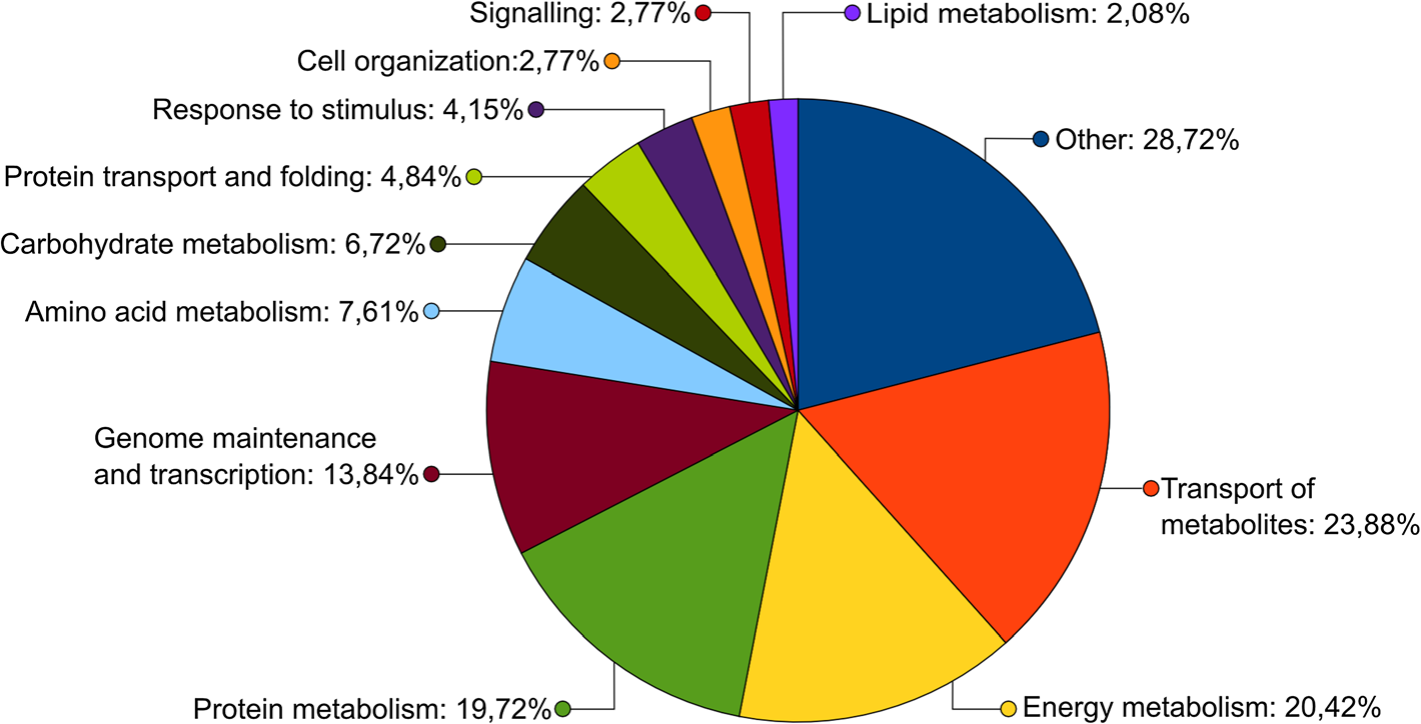
**Distribution of Gene Ontology (GO) categories for the *C*. *cacaofunesta *nuclear genes encoding the mitochondrial proteins that were identified using LC**-**MS/MS**.

The same GO categorization was performed using the experimental proteome. For this purpose, the 388 proteins with potential mitochondrial localizations that had been identified using LC-MS/MS were subjected to a GO analysis (Additional file 6, Sheet 6A). The results shown in Figure 5 revealed that the categories most strongly represented in the experimental proteome were transport of metabolites (23.9%), energy metabolism (20.4%) and protein metabolism (19.7%). The classifications of the experimental proteins reflect the *C. cacaofunesta* culture conditions (malt, yeast extract and agar). The fungus grows rapidly, suggesting an intense metabolism, including the synthesis of enzymes and the import of proteins and metabolites. Moreover, the availability of glucose as the primary carbon source likely enhanced the expression of genes that are involved in energy metabolism. Vodisch and coworkers (2011) examined the mtProt of *Aspergillus nidulans* as a part of the global fungal proteome [76]. These authors observed a clear relationship between the culture conditions and the enriched classes of experimentally identified proteins.

Notably, the global analysis of the *C. cacaofunesta* mitochondrial function that was based on functional classification (GO) of the experimentally identified proteins (Figure 5) reflects the functional grouping of the full set of predicted mitochondrial proteins (Figure 4). Considering that we performed mass spectrometry only for fungi in cultured conditions, which appears to be far from mimicking fungal growth in nature, the similarity of functions between the predicted and experimental proteomes reinforces the importance of the core mitochondrial functions. Additionally, this similarity suggests that the mitochondrial role in the adaptation of the fungus to certain growth conditions, including the environment of its host, depends of specific sets of proteins, pathways and/or differential gene regulation rather than on major changes in mitochondrial function.

### *C. cacaofunesta* global proteome survey

It is well established that mitochondrial function is important for the virulence and pathogenicity of fungi [16,77]. Here, we demonstrated that the global proteome compositions of *S. cerevisiae*, *N. crassa* and *C. cacaofunesta* do not contain major differences. However, of these three fungi, *C. cacaofunesta* is the only pathogenic fungus, which prompted us to perform a more detailed investigation of its proteome composition.

Early stages of necrotrophic infections, like those caused by *C. cacaofunesta*, are associated with host cell death, the production of secondary metabolites and the accumulation of reactive oxygen species (ROS) [78,79]. Mitochondrial proteins that are associated with detoxification may play an important role in the success of pathogen colonization. We therefore analyzed the predicted mtProt of *C. cacaofunesta* to identify proteins that may play important roles in pathogenicity and sensitivity/resistance to fungicides.

**Figure 6:**
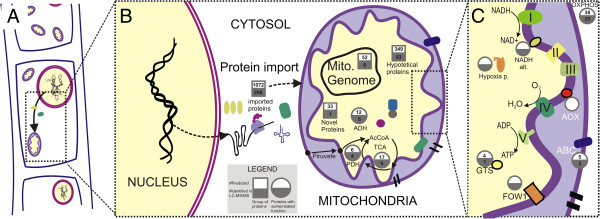
**Schematic summary of the *C*. *cacaofunesta *mitochondrial proteome survey.** (**A**) Detailed representation of a hypha, including the nucleus and the mitochondria. (**B**) Nuclear – mitochondrial communication represented by the protein import (predicted/identified by LC-MS/MS). In the mitochondria are represented proteins involved in pyruvate dehydrogenase complex (PDH) and tricarboxylic acids cycle (TCA). Also, are represented aldehyde dehydrogenase (ADH); hypothetical proteins; novel proteins and proteins encoded in mtDNA. (**C**) Zoom of the mitochondrial subcompartments. Proteins involved in oxidative phosphorylation (OXPHOS) and polypeptides with probable role in pathogenicity are depicted. Cytochrome-dependent respiratory chain components (complexes I to V); alternative respiratory pathway including alternative NADH (NADH alt) and alternative oxidase (AOX); glutathione S-transferases (GST); ATP-binding cassette transporters (ABC) and protein homologous to mitochondrial carrier of *Fussarium oxysporum* (FOW1).

We focused the search on proteins that have been previously related to virulence, specifically ATP-binding cassette (ATP) transporters, aldehyde dehydrogenase, alternative NADH dehydrogenase, alternative oxidase, the mitochondrial carrier FOW1, glutathione S-transferase and hypoxia-related protein. Figure 6 shows a schematic summary of a subset of the predicted proteins in the mtProt of *C. cacaofunesta* with potential involvement in the pathogenicity of this fungus.

The *C. cacaofunesta* predicted mtProt contains 5 proteins classified as ABC transporters. All of the genes codifying these proteins were expressed under the tested conditions, with average RPKM values of 140.6 and 1,463.7. Mass spectrometry identified 2 of this class of transporters (Additional file 2, Sheet 2B; Additional file 4, Sheet 4A). The ABC transporter of a *Nectria haematococca* (NhABC1) proved to be an important virulence factor that contributes to the *N. haematococca* tolerance of phytoalexin during its interaction with the host [79]. Furthermore, ABC transporters have been associated with fungicide resistance [80].

Aldehyde dehydrogenase (ADH) has also been implicated in the pathogenicity of fungi. ADH is regarded as a detoxification enzyme due to its role in the metabolism of intermediates and exogenous aldehydes [81]. An analysis of *adh* expression in *Clasdosporium fulvum,* both *in vitro* and *in vivo*, during its interactions with tomato indicated that the expression of this gene was associated with infection and starvation conditions [82]. Interestingly, the predicted *C. cacaofunesta* mtProt survey identified 12 ADH-encoding genes with confirmed expression (RPKMs > 10), and 6 ADH proteins were identified by LC-MS/MS. Moreover, glutathione S-transferases (GSTs) are a large family of proteins that are involved in cellular detoxification processes during oxidative stress responses [83] and may be involved in fungicide resistance [84]. Mao and coworkers (2012) suggested a link between the number of GST-encoding genes in the fungal genome and its lifecycle [85]. The predicted *C. cacaofunesta* mtProt contains 4 proteins that are homologous to GST. The RNA-Seq analysis indicated varying average expression levels, with RPKM values of as high as 150; one of those proteins was identified in the experimental proteome (Additional file 2, Sheet 2B; Additional file 4, Sheet 4B).

Moreover, components of the alternative respiratory chain have been related to fungal pathogenicity, specifically alternative NADH dehydrogenase [86] and alternative oxidase (AOX) [87]. Studies in which alternative NADH dehydrogenase was blocked in *Aspergillus niger* demonstrated the role of this protein in protection against oxidative stress [88]. *C. cacaofunesta* has one encoded alternative NADH dehydrogenase gene with RPKM values > 60; this gene was also identified in the experimental proteome (Additional file 2, Sheet 2B; Additional file 4, Sheet 4B). Moreover, the predicted mtProt included a putative AOX. This enzyme has been implicated in fungal protection against oxidative stress, pathogenicity and fungicide resistance [89,90]. *C. cacaofunesta* AOX was not identified in the experimental proteome, but the gene *aox* exhibited detectable expression, with a RPKM value of approximately 88 (Additional file 2, Sheet 2B; Additional file 4, Sheet 4A). Recently, Thomazella and coworkers (2012) provided strong evidence for the involvement of AOX in maintaining the infective phase of the basidiomycete *Moniliophthora perniciosa*[91].

The deletion of a *Fusarium oxysporum* mitochondrial carrier-encoding gene (*fow*1) caused the loss of its ability to infect plant tissues [92]. A putative FOW1 protein was identified in the *C. cacaofunesta* predicted mtProt. The RNA-Seq data indicated that the FOW1 homologue in *C. cacaofunesta* was highly expressed (with an RPKM value of 536), and its product was identified in the experimental proteome (Additional file 2, Sheet 2B; Additional file 4, Sheet 4A).

It has been reported that the ability of certain fungi to withstand different levels of hypoxia is a critical virulence factor in terms of fungal pathogenicity in mammals [93]. Hypoxia-related genes have been identified as important virulence factors for *Aspergillus fumigatus* and *Candida albicans* given that they are exposed to low oxygen concentrations during infection [93,94]. Oxygen levels in the secondary xylem, the plant tissue that is infected by *C. cacaofunesta*, are as low as are observed in human tissues that are infected by these fungal pathogens, reaching levels less than 5% [93,95]. This observation suggests *C. cacaofunesta* should possess mechanism with which to adapt to hypoxia. In this regard, the predicted *C. cacaofunesta* mtProt contains one protein that has a conserved hypoxia response domain. This gene was expressed in *in vitro* conditions, with an RPKM value as high as 259 and its product was also identified in the experimental proteome (Additional file 2, Sheet 2B; Additional file 4, Sheet 4A). Although hypoxia response mechanisms are more complex and involve a series of regulatory pathways [96], these results open the way for the exploration of the role of *C. cacaofunesta* mitochondria in fungal phytopathogens responses to hypoxia.

Due to inherent limitations for each of the techniques that were used to predict the subcellular localization of proteins, we chose to perform a stringent integrative approach to predict the *C. cacaofunesta* mtProt. We therefore must state that the list of mitochondrial proteins proposed here as the predicted mitochondrial proteome of *C. cacaofunesta* may not correspond to the full set of mitochondrial polypeptides. This mtProt survey is a preliminary analysis due to the relatively small number of proteins discussed but reveals how these data can be exploited in future studies. The presented genome and estimated proteome data provide a starting point for the improved characterization of *C. cacaofunesta* mitochondrial function in two ways: (i) facilitating the identification of proteins that may be relevant to the lifestyle of this fungus and (ii) expanding the literature that relates to studies of mitochondrial function and evolution to the discovery of new mitochondrial genes. In this regard, the identification of both conserved proteins with unknown function and novel proteins is particularly relevant. The comprehensive characterization of these proteins opens up exciting new opportunities for understanding the role of mitochondria in phytopathogenicity. Due to the important role of this organelle in resistance/susceptibility to fungicides, this study could aid in the identification of more effective strategies by which to the control this fungus.

## Conclusions

In the present study, we performed a global analysis of *C. cacaofunesta* mitochondria using an integrative approach (mitogenomics, transcriptomics and proteomics). We predicted the *C. cacaofunesta* mitochondrial proteome, including 52 mitochondrial encoded proteins and 1,072 proteins encoded in the nuclear genome (NMP). All of the NMP (except for 10 genes) were transcribed in mycelia that were grown *in vitro*; approximately 27% of these genes were also identified by mass spectrometry. RNAseq analysis allowed to detected expression of 33 novel putative genes. The comparison of the global analysis of mitochondrial function between *C. cacaofunesta*, *S. cerevisiae* and *N. crassa* revealed that, in addition to the known involvement of mitochondria in the pathogenesis of fungi, the differences in the lifestyle of these organisms (pathogenic and no-pathogenic) are not accompanied by major differences in the functional composition of their mitochondrial proteomes. Therefore, the relevance of this organelle in different fungi lifestyle could lie in a particular set of proteins and/or in a different pattern of gene regulation. In this regard, LC-MS analysis validated the existence of 84 proteins with unknown function, of which one is probably specific to this species and likely to the *Ceratocystis* species complex. Further functional characterization of these putative mitochondrial proteins could improve our understanding of the mitochondria’s role in fungal pathogenesis.

## Methods

### Biological material and nucleic acid isolation

Dr. Tomas Harrington, from the Iowa State University Department of Plant Pathology, generously donated *Ceratocystis cacaofunesta* strain C1593. This strain was isolated in 1999 from infected cacao trees located on a farm in the district of Uruçuca, Bahia, Brazil. In our laboratory, *C. cacaofunesta* cultures were maintained on 2% malt, yeast extract and agar (MYEA) plates inside a BOD chamber at 28°C. For liquid cultures, 20 to 30 agar blocks (1 mm^2^) that were removed from the solid cultures were inoculated into flasks that contained 100 mL of malt media and were cultivated at 28°C for 7 days, under constant agitation at 150 rpm.

The mycelia were separated from the media using filtration, were washed twice with sterile distilled water and were frozen with liquid nitrogen. The samples were ground to a fine powder in a mortar and were processed for DNA isolation as previously described [97]. The RNA isolation was performed using the RNeasy Plant Mini Kit (Qiagen, Valencia, CA, USA), according to the manufacturer’s protocol. All of the isolated genetic materials were qualitatively analyzed using denaturing formaldehyde/agarose gel electrophoresis and were quantified using a NanoDropTM 1000 spectrophotometer (Thermo Scientific).

### Genome sequencing and assembly

The *C. cacaofunesta* mitochondrial genome was sequenced as part of the *C. cacaofunesta* Genome Project (http://www.lge.ibi.unicamp.br/ceratocystis). The DNA was sequenced on a Genome Analyzer IIx platform (Illumina) at the University of North Carolina, Chapel Hill High-Throughput Sequencing Facility. The whole-genome shotgun strategy was used to produce 76-bp paired-end reads (400-bp insert size) and 36-bp mate-pair reads (3-kb insert size).

The paired-end reads were assembled into longer scaffolds using *de novo* assembler VELVET 1.0.12 software [98] with a k-mer parameter of 69, which maximizes the length of the mitochondrial DNA (mtDNA) contig. The mtDNA was identified through comparisons with *C. cacaofunesta* scaffolds and *Gibberella zeae* mtDNA (NC_009493), resulting in the identification of a single mtDNA contig. To verify the topology of the mtDNA, we used the paired-end and mate-pair reads that had been aligned with the mtDNA using the SOAP2 aligner [99]. The reads that mapped to the borders of the contig were located at an expected distance from their respective pairs.

The complete mtDNA sequence was deposited into the GenBank database and is available under the accession number JX185564.

### mtDNA annotation

*C. cacaofunesta* mtDNA was inspected by tBLASTn searches to identify 14 known conserved coding genes using their *Gibberella zeae* mtDNA orthologous as the query (Accession NC_009493). The *rps3* gene was predicted using the *C. fimbriata rps3* sequence available at GenBank (Accession FJ895616.1). Open Reading Frame Finder (ORF Finder) was used to search for intronic and hypothetical ORFs using Genetic code 4 (mold mitochondrial). Alternative start codons were allowed for predicted intronic ORFs when the initial ATG site resulted in an incomplete LAGLIDADG or GIY-YIG domain as indicated by a BLASTp search. The set of tRNAs was identified using tRNAscan-SE [48]. Genes encoding both small and large rRNA subunits were identified through comparisons with orthologous *Gibberella zeae* sequences using BLAST2seq (BLASTn).

### mtDNA GC content and GC skew

The GC content, local GC content and cumulative GC skew of the *C. cacaofunesta* mtDNA were calculated using customized Perl scripts (available upon request). For the local GC content and cumulative GC skew, a sliding window of over 5,000 bp with a 500-bp range was used.

### Predicted mitochondrial proteome

The putative genes encoding NMPs were identified using two strategies: *ab initio* prediction and sequence comparison. For the *ab initio* prediction, SignalP 3.0 software [100] was used to analyze all of the predicted nuclear proteins to identify those with a low probability (≤ 50%) of containing a signal peptide. Using the SignalP results as the input, the WoLF PSORT [101] and TargetP [100] programs were used to classify putative mitochondrial proteins. Within the TargetP output, only the proteins that were identified with a high probability of encoding mitochondrial genes (mTP ≥ 50% and other ≤ 50%) were considered. The proteins that were identified by both programs (TargetP and WoLF PSORT) constituted the final *ab initio* protein dataset. For the sequence comparison approach, we built a database that contained 1,583 known fungal mitochondrial proteins (Additional file 7; 741 proteins from *S. cerevisiae* and 842 from *N. crassa*). After the *C. cacaofunesta* nuclear encoded proteins were compared against this database using BLASTp (with an e-value cutoff of 1e^-5^), the comparative protein dataset was obtained from the BLAST results by applying query coverage (≥ 70%) and similarity (≥ 70%) filters. The *ab initio* and comparative protein datasets were merged to generate the final NMP dataset. The sequences of the nuclear-encoded mitochondrial proteins from *C. cacaofunesta* are provided in Additional file 8.

The automatic annotation of the NMPs was performed using the NR/NCBI, KEGG [62] and UniRef90 databases (BLASTp with an e-value cutoff of 1e^-5^) [61] and summarized using the AutoFACT program [60]. The CDD/Pfam database was used to identify the conserved domains. The Blast2GO program [64] was used to perform the gene ontology classification (BLASTp, with an e-value cutoff of 1e^-5^ on Generic GO Slim; the first 50 hits were considered). The proteins with identified biological functions according to GO Slim were manually grouped into 12 classes: amino acid metabolism, carbohydrate metabolism, cell organization, energy metabolism, genome maintenance and transcription, lipid metabolism, protein metabolism, protein transport and folding, response to stimulus, signaling, transport of metabolites and other. The proteins that were classified into multiple categories were considered to be members of both categories, with the exception of proteins that were classified as both “other” and another category; these were excluded from the “other” category and remained in the specific category. Thus, the “other” category includes proteins of known function that were not included in any of the above-described categories. The GO Slim biological processes that clustered in each category are shown in Additional file 5. Similar analyses were performed for the *S. cerevisiae* and *N. crassa* mitochondrial proteomes to compare them with the *C. cacaofunesta* data (Additional file 6, Sheets 6A, 6B and 6C).

### Preparation of RNA-seq libraries and transcriptome analysis

RNA from *C. cacaofunesta* liquid cultures was extracted using the RNeasy Plant mini kit (Qiagen). Approximately 5 μg total RNA were used to prepare RNA-seq libraries following the procedures described by the manufacturer (Illumina). Briefly, the mRNA was purified using Sera-mag oligo (dT) beads and subsequently used for cDNA synthesis. Double-stranded cDNA was subjected to end-repair, A-tailing, adapter ligation and PCR amplification. The libraries were quantified using a Qubit fluorometer (Invitrogen), and quality control was performed using the Experion automated electrophoresis system (Bio-Rad). Two independent biological replicates were used for transcriptome sequencing. Each sample was sequenced in one lane of an Illumina Genome Analyzer IIx sequencer. Approximately 27 and 29 million single-end reads of 36 bp were produced for the CER1 and CER2 libraries, respectively.

The SOAP2 aligner [99] was used to align the RNA-Seq reads from mycelia that were grown *in vitro* with predicted genes that encode nuclear-encoded mitochondrial proteins. The program was configured to allow for as many as two mismatches, to discard sequences that contained “N”s and to return all optimal alignments. The expression level of each gene was estimated as an RPKM (reads per kilobase of exon per million reads mapped) value [102].

### Mitochondrial isolation and proteomic assays

#### Mitochondrial isolation

The mitochondrial isolation was performed according to the protocol that is described by Sorensen and coworkers [103], with minor modifications. *C. cacaofunesta* was cultured for seven days in standard MYEA growth medium. Subsequently, the fungal mycelia were washed in cold water and homogenized using a bead beater in cold extraction buffer that contained 330 mM sucrose, 10 mM Tris–HCl, 1 mM EDTA, 1% PVP, 0.1% BSA and 0.3 mM PMSF (pH 7.5). The homogenates were filtered and centrifuged at 1,500 g for 15 min to remove the cellular debris. The resulting supernatants were centrifuged at 15,500 g for 20 min. The samples were rinsed twice in wash buffer (330 mM sucrose, 10 mM Tris–HCl, 1 mM EDTA, pH 7.2), and the final mitochondrial preparation was used to perform the proteomic assays.

#### The identification of proteins using LC-MS/MS

##### *Enzymatic in-gel digestion for mass spectrometry analysis*

A total of 20 μg of the concentrated mitochondrial proteome was obtained as described above and was separated using one-dimensional SDS-PAGE electrophoresis. Nineteen bands were excised from the gel and subjected to in-gel trypsin digestion, as described previously [104], with modifications.

The resulting peptide solution was dried in a SpeedVac concentrator and resuspended in 100 μL of 0.1% formic acid. An aliquot of 4.5 μL was separated using C18 (75 μm × 100 mm) RP-nanoUPLC (nanoACQUITY, Waters) coupled with a Q-Tof Ultima mass spectrometer (Waters) with a nano-electrospray source. The flow rate was 600 nL/min, and the gradient was 2-90% acetonitrile in 0.1% formic acid over 45 min. The instrument was operated in the “top three” mode, in which one MS spectrum was acquired, followed by an MS/MS analysis of the three most intense peaks [105].

The spectra were acquired using MassLynx v.4.1 software, and the raw data files were converted to a peak list format (mgf) using the Mascot Distiller v.2.3.2.0, 2009 software (Matrix Science Ltd.) without summing the scans, allowing for a label-free analysis (Additional file 9). The files were then searched against the *Ceratocystis cacaofunesta* database (7,321 entries – 7,269 nuclear proteins, and 52 mitochondrial proteins) using Mascot engine v.2.3.01 (Matrix Science Ltd.). Carbamidomethylation was used as a fixed modification, methionine oxidation as a variable modification, one missed trypsin cleavage and a tolerance of 0.1 Da for both precursor and fragment ions. For the protein quantitation, the .dat files from the Mascot output were analyzed using Scaffold Q+ (version 3_00_03, Proteome Software), and quantitative values (normalized spectral counts) were obtained [106,107]. For the endogenous peptide identification, methionine oxidation was set as a variable modification, with a tolerance of 0.1 Da for both the precursor and fragment ions. Only peptides with a minimum of 5 amino acid residues and significance (p < 0.05) based on the Mascot-based scores were considered in the results.

## Abbreviations

ABC tranporter: ATP binding cassette (family of membrane transport proteins); ADH: Alcohol dehydrogenase; AOX: Alternative oxidase; ATP: Adenosine triphosphate; BLAST: Basic local alignment search tool; BSA: Bovine serum albumin; CDD: Conserved domains database; COB: Cytochrome b oxidade; COX: Cytochrome c oxidase; CS: Citrate synthase; EDTA: Ethylenediaminetetraacetic acid; FOW1: Fusarium oxysporum mitochondrial carrier; GO: Gene ontology; GST: Glutathione S-transferase; IDH: Isocitrate dehydrogenases (IDH1 and IDH2); KEGG: Kyoto encyclopedia of genes and genomes; NAD: Nicotinamide adenine dinucleotide (electron donor); NADH: Reduced B-nicotinamide adenine dinucleotide; NCBI: National Center for Biotechnology Information; NMP: Nuclear-encoded mitochondrial proteins; NR: Non-redundant GenBank database; ORF: Open reading frame; OXPHOS: Proteins of oxidative phosphorylation machinery; PDH: Proteins of the pyruvate dehydrogenase complex (; PMSF: Phenylmethylsulfonyl fluoride; PVP: Polyvinylpyrrolidone; rnl: Large RNA subunit; rns: Small RNA subunit; RPKM: Reads per kilobase of exons per million; rpo: RNA polymerase; SDS-PAGE: Sodium dodecyl sulfate polyacrylamide gel electrophoresis; TCA: Tricarboxylic acid cycle; Tris–HCl: Tris (hydroxymethyl) aminomethane hydrochloride; UFP: Unknown function protein

## Competing interests

The authors declare that they have no competing interests.

## Authors’ contributions

ABA: preparation of the manuscript and data analysis; LCN, MFC, ROV: bioinformatics analysis and revision of manuscript; BVO, DPTT: collaboration in sampling the material and the revision of manuscript; RAT: collaboration in bioinformatics analysis; AFPL: collaboration in the generation of LC-MS/MS data; PJPLT, PM: sequencing of the libraries by RNAseq, and collaboration in data analysis; LWM: collaboration in sampling the material; GAGP, OGC: coordination of the molecular and bioinformatics analysis, preparation and organization of the manuscript. All of the authors have read and approved the final version of the manuscript.

## Supplementary Material

Additional file 1 All intronic ORFs sequences. Click here for file

Additional file 2 **Table of all genes predicted in this work and its RPKMs and its annotations (RPKMs, NR, KEGG, CDD, Autofact, Biological process, Molecular function and Cellular component, Predicted by BlastP, Predicted by TargetP/WolfpSort, Identified by LC-MS/MS).** Sheet 2A: mitochondrial genes. Sheet 2B: predicted nuclear genes. Click here for file

Additional file 3 Scheme of experimental method. Click here for file

Additional file 4 **Table of all LC-MS/MS.data.** Sheet 4A: all proteins identified by LC-MS/MS. Sheet 4B: new annotation proposed to hypothetical proteins. Sheet 4C: possible cellular location of proteins not predicted. Click here for file

Additional file 5 Table grouping of biological process categories identified by GOslim. Click here for file

Additional file 6 Table of mitochondrial predicted genes annotation by GOslim. Click here for file

Additional file 7 Amino acid sequences of fungal mitochondrial proteins. Click here for file

Additional file 8 Amino acid sequences of all proteins analyzed in this work. Click here for file

Additional file 9 Table mzML of mass spectrometry data. Click here for file
