# Supplementary figures and images for: Global analyses of Ceratocystis cacaofunesta mitochondria: from genome to proteome
Source: BMC Genomics. 2013 Feb 11;14:91. doi: 10.1186/1471-2164-14-91 (PMC3605234; doi:10.1186/1471-2164-14-91)

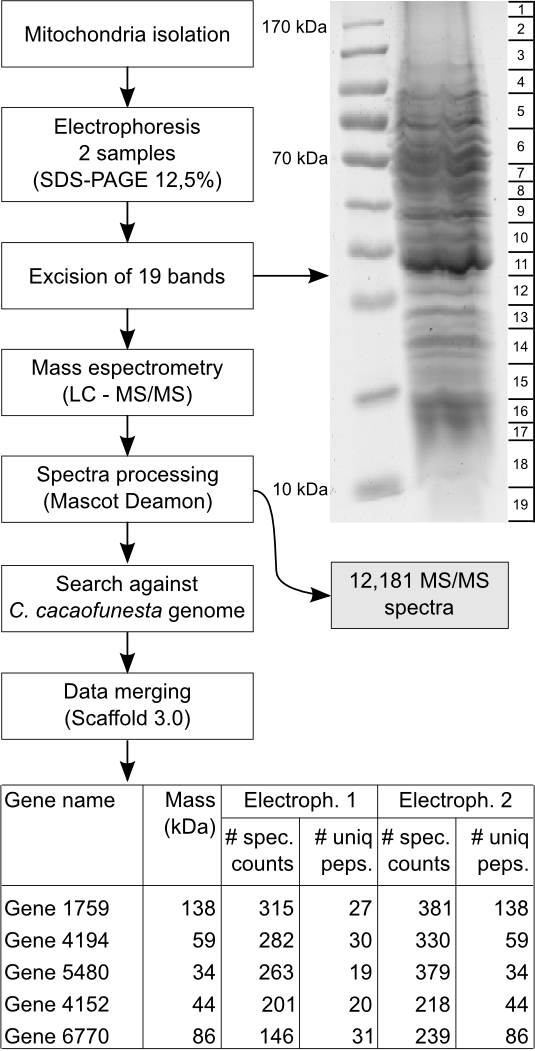

Supplement: Additional file 3 — Scheme of experimental method. [file 1471-2164-14-91-S3.png]
